# Speciation and transformation of nitrogen for swine manure thermochemical liquefaction

**DOI:** 10.1038/s41598-022-16101-w

**Published:** 2022-07-14

**Authors:** Zhuangzhuang Liu, Zhiwei Yan, Fen Liu, Jun Fang

**Affiliations:** 1 College of Bioscience and Biotechnology, Hunan Agricultural University, Changsha, 410128 Hunan People’s Republic of China; 2 Hunan Engineering Laboratory for Pollution Control and Waste Utilization in Swine Production, Changsha, 410128 People’s Republic of China

**Keywords:** Energy science and technology, Renewable energy, Bioenergy, Biofuels

## Abstract

The nitrogen conversion mechanism of swine manure by thermochemical liquefaction with ethanol as solvent was investigated at a lower temperature range (180–300 °C). The fate of nitrogen in liquid phase products, bio-oil and biochar was evaluated by XPS, GC–MS and other methods. After thermochemical liquefaction, most of the nitrogen in swine manure was transferred to biochar (63.75%). As the temperature increased to 220 °C, the biochar-N yields decreased to 43.29%, accompanied by an increase in bio-oil-N and liquid phase product-N by 7.99% and 1.26% respectively. The results indicated that increasing the temperature could facilitate solid nitrogen structure cracking into bio-oil-N. Amines and heterocyclic nitrogen from protein peptide bond cracking and Maillard reactions made up the main nitrogen compounds in bio-oil, and high temperatures favored the further cyclization and condensation of heterocyclic nitrogen (e.g., indole, quinoline). In the case of biochar, the inorganic nitrogen disappeared at 260 °C and was obviously transformed into liquid phase products. The rising temperature promoted the polymerization of pyridine nitrogen and pyrrole nitrogen, which formed more stabilized nitrogen formation (such as quaternary nitrogen). Nitrogen conversion and possible reaction schematics during swine manure thermochemical liquefaction were explored in this study.

## Introduction

With the continuous improvement and development of the livestock and poultry breeding industry in China, the rapid increase of livestock pollutants such as swine manure and cow manure, has generated a great challenge in the field of related waste disposal^1^. According to the Chinese National Bureau of Statistics (NBSC, 2019)^2^, over 3.8 billion tons of livestock manure are produced from large-scale breed farms every year, 38.34% of which are swine manure (SM). SM contains massive potassium, phosphorus and nitrogen. These organic nutrients are essential for modern agriculture and fertilizer production, but they also have potential harmful effects on farmland due to excessive heavy metal elements (Cu, Zn, As, Cr etc.) and salts^3^. Further, pathogenic bacteria (*Salmonella spp*., *Escherichia coli*, and *Campylobacter* spp.) and undegraded antibiotics (tetracyclines) are enriched in untreated SM, which poses serious threats to environment^4,5^. Composting is a classic way to deal with SM, but previous studies show that the unequal portion of nitrogen in this livestock waste greatly exceeds the demand of plants and agricultural land, which leads to an excess accumulation of nitrates^6,7^. The other disadvantages of composting are the long fermentation period and nitrogen loss, which are easily affected by weather and season. Notably, rainwater takes this nitrogen into soils, underground water and other water bodies, resulting in algae pollution^8^. Therefore, a technology for the comprehensive treatment and resource utilization of SM is urgently needed.

From the point of view of energy, SM is considered a potential raw material for producing biofuels and the application of biofuels is relatively mature^9,10^. Furthermore, as a new generation of biomass, SM contains high hydrocarbon content, and the higher heating value is a little higher than that of livestock manure, which is appropriate for thermochemical utilization^11^. Lu et al.^12^ compared bio-oil yields produced from livestock manures via hydrothermal liquefaction and indicated that SM had the highest yield of bio-oil (30.8%). Sharara et al.^13^ investigated various bioenergy conversion techniques of SM and evaluated thermochemical liquefaction as a suitable technique to process SM. Thermochemical liquefaction is a promising and environmentally friendly technology that has received considerable critical attention in recent years^14^. Unlike combustion, which releases hazardous products, biomass rapidly oxidizes or mineralizes to form innocuous substances under thermochemical liquefaction conditions. The system designed by KS et al. could process 1.5 tons of SM per month and efficiently produce bio-oil under the liquefaction process^15^. Its attractive features include: (1) effective transformation of organic compounds in biomass into bio-oil and biochar materials without pretreatment^16^ and (2) conversion of most residual heavy metals in SM into solid residue, with complete inactivation of antibiotic-resistant genes after thermochemical liquefaction^17^. However, most studies have only focused on exploring the optimum reaction parameters (temperature, solvent, catalyst, etc.) for bio-oil and biochar^18,19^. The transformation and pathways of nitrogen migration in thermochemical liquefaction products from SM have seldom been reported. Determining how nitrogen is transformed and evolved should be concurrently investigated with finding the most proper reaction temperature and ensure a lower energy consumption.

Several studies have indicated that the fate of nitrogen during the liquefaction of biomass relies heavily on the severity of the reaction condition and on the type of feedstock^20^. Xiao et al.^21^ found that increasing temperature promoted more nitrogen migration from biochar to the bio-oil. The main N-containing compounds detected in bio-oil were amine, nitrile, and N-heterocyclic compounds. Zhang et al.^22^ suggested that nitrogen in microalgae during liquefaction was divided into inorganic-N (5%) and protein-N (95%). The protein was hydrolyzed into amino acids and was responsible for the formation of N-heterocyclic compounds and pyrazines in the bio-oil. The inorganic-N and unstable protein-N were decomposed and transformed into solid products. Moreover, the quality of bio-oil and biochar from thermochemical liquefaction is strongly linked with nitrogen content^23^. Unfavorable nitrogenous compound production such as recalcitrant N-heterocyclic compounds, also results in resource and energy losses. Further, N_1_ compounds are more refractory to hydrotreatment than oxygen and nitrogen heteroatomic compounds, and other nitrogen compounds with methyl groups on their rings express severe toxicity on the mammal cells^24,25^. As for biochar, the main solid product during thermochemical liquefaction, it is considered a promising raw material for solid fuels and carbon fuel cells^26,27^. However, there is a large amount of nitrogen from pig manure in biochar, which is converted into NOx and discharged into the atmosphere, causing an undesirable burden on the nitrogen cycle of the ecological environment. Hence, understanding the evolution and fate of nitrogen in all products is essential for the future industrialization of thermochemical liquefaction.

In this study, we focused on the speciation and transformation of nitrogen during thermochemical liquefaction of SM at temperatures between 180 and 300 °C. The main objectives are threefold: (1) investigating the nitrogen distribution in liquid phase products, bio-oil, and biochar; (2) evaluating the nitrogen compounds in bio-oil, nitrogen evolution in biochar, and nitrogen transformation in liquid phase products; and (3) exploring possible nitrogen conversion mechanisms of SM during thermochemical liquefaction. This study provides new insights into the application of SM as a renewable energy resource and proposes a reaction pathway for nitrogen during thermochemical liquefaction.

## Results and discussion

### Mass balance of N in products during thermochemical liquefaction at different temperatures

**Figure 1:**
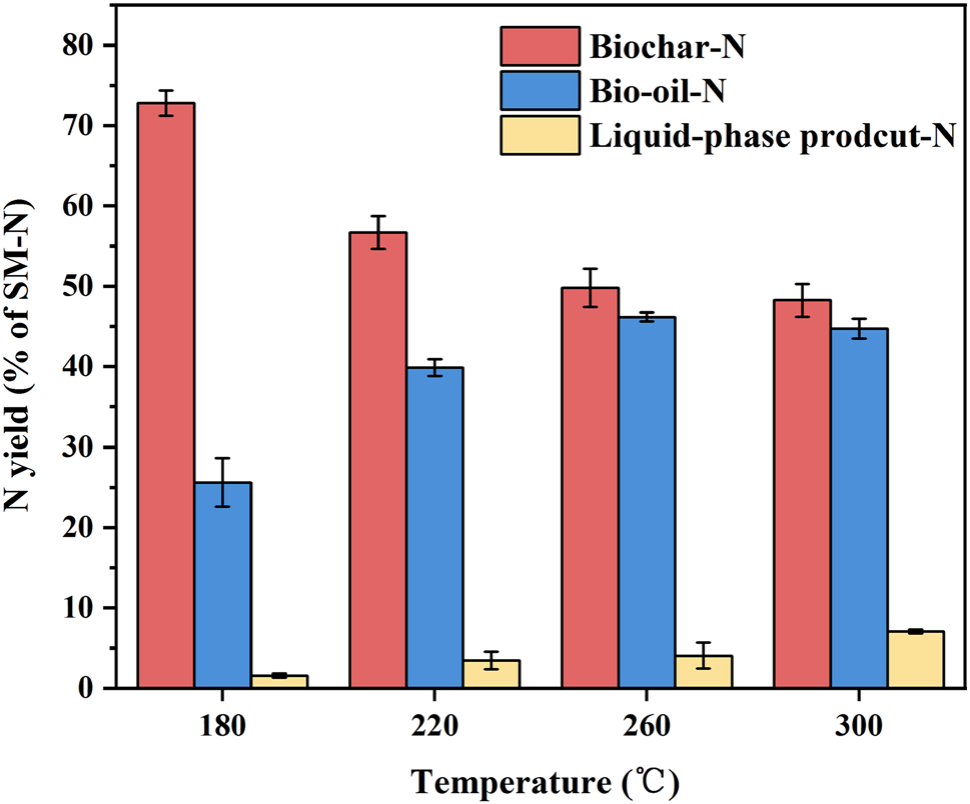
Mass balance of N in products during thermochemical liquefaction at different temperatures.

The mass balance of nitrogen in products during thermochemical liquefaction at different temperatures is shown in Fig. 1, and the specific values can be seen in Table 1. We observed that most of the nitrogen in SM was transferred to the biochar at 180 °C (72.8%, 195 mg), whereas there was no remarkable change in biochar nitrogen at the reaction temperature between 220 and 300 °C. Based on the evolution of nitrogen in different phases, a two-stage process of nitrogen distribution against the reaction temperature during the thermochemical liquefication of SM was suggested. In the initial reaction temperature range (180–220 °C), the biochar-N yields showed a sharp decrease from 72.8% (195 mg) to 56.7% (152 mg), accompanied by the bio-oil-N and liquid phase product-N increased by 14.3% (38 mg) and 1.8% (5 mg), respectively. The results indicated that increasing the temperature facilitated solid nitrogen structure cracking into bio-oil-N. Furthermore, the cracking of biochar-N generated a small amount of aqueous compounds. Furthermore, the increase in bio-oil-N may be due to the use of ethanol as a solvent during thermochemical liquefaction, which could enhance the hydrolysis of macromolecular organics in the liquid phase^28^. In the second stage (above 220 °C), the biochar-N showed a minor decline at 260 °C (49.7%, 133 mg) but stabilized at 300 °C (48.3%, 129 mg), despite a low yield. Meanwhile, the liquid-phase product-N increased. This was because the polymerization of nitrogen-containing compounds was enhanced under a high reaction temperature, which led to biochar-N dissolving into the liquid phase product^29^. These results are consistent with the literature^30^. Additionally, compared to swine manure, liquefied bio-oil has higher HHV in the range of 30–39 MJ/kg due to the low O/C and high H/C ratios (Table A, supplementary). This suggests that the presence of ethanol promoted the hydrodeoxygenation reaction. The HHV of all bio-oil samples increased with elevated temperature, which also enhanced the hydrogenation and decarboxylation processes, allowing the bio-oil to be upgraded more efficiently.

**Table 1:** The mass balance of N in products during thermochemical liquefaction.

Sample	Mass of feedstock/g	Mass of N/mg^a^			
		TN^b^	Biochar—N^c^	Bio-oil—N^c^	Liquid—N^d^
**Temperature range**					
180 °C	10.001	268	195 (72.8%)	69 (25.6%)	4 (1.6%)
220 °C	10.002	268	152 (56.7%)	107 (39.9%)	9 (3.4%)
260 °C	10.003	268	133 (49.7%)	124 (46.2%)	11 (4.1%)
300 °C	10.002	268	129 (48.3%)	118 (44.7%)	21 (7.0%)

^a^The value in brackets represents the fraction of phase-N in total-N.

^b^Total mass of N was calculated from the N fraction in SM (2.68%, as shown in Table 3) and the mass of the feedstock used.

^c^Total mass of N in solid/oil was calculated from the N fraction in solid/oil and the mass of solid/oil, respectively.

^d^The mass of N in liquid was calculated from the TN-N in the liquid and the volume of the hydrolysate.

The bio-oil-N yield exhibited an intense increase from 220 °C (39.9%, 107 mg) to 300 °C (44.7%, 118 mg). Meanwhile, as the reaction temperature increased, the liquid phase product-N yield showed the same trend as the bio-oil-N yield, which reached 4.1% (11 mg) at 260 °C, becoming steady at 300 °C (7.0%, 21 mg). It could be deduced that when the reaction temperature was under a certain range (260–300 °C), the slight fluctuation of nitrogen between the liquid and oil phase was mainly due to the unattainable equilibrium level between the hydrolysis reaction and the compound reaction^31^. As mentioned above, bio-oil-N was much higher than biochar-N and liquid phase product-N during thermochemical liquefaction. Less nitrogen was released from biochar into the bio-oil or liquid phase product in the second temperature range.

### Effect of thermochemical liquefaction on N transformation in liquid phase products

**Figure 2:**
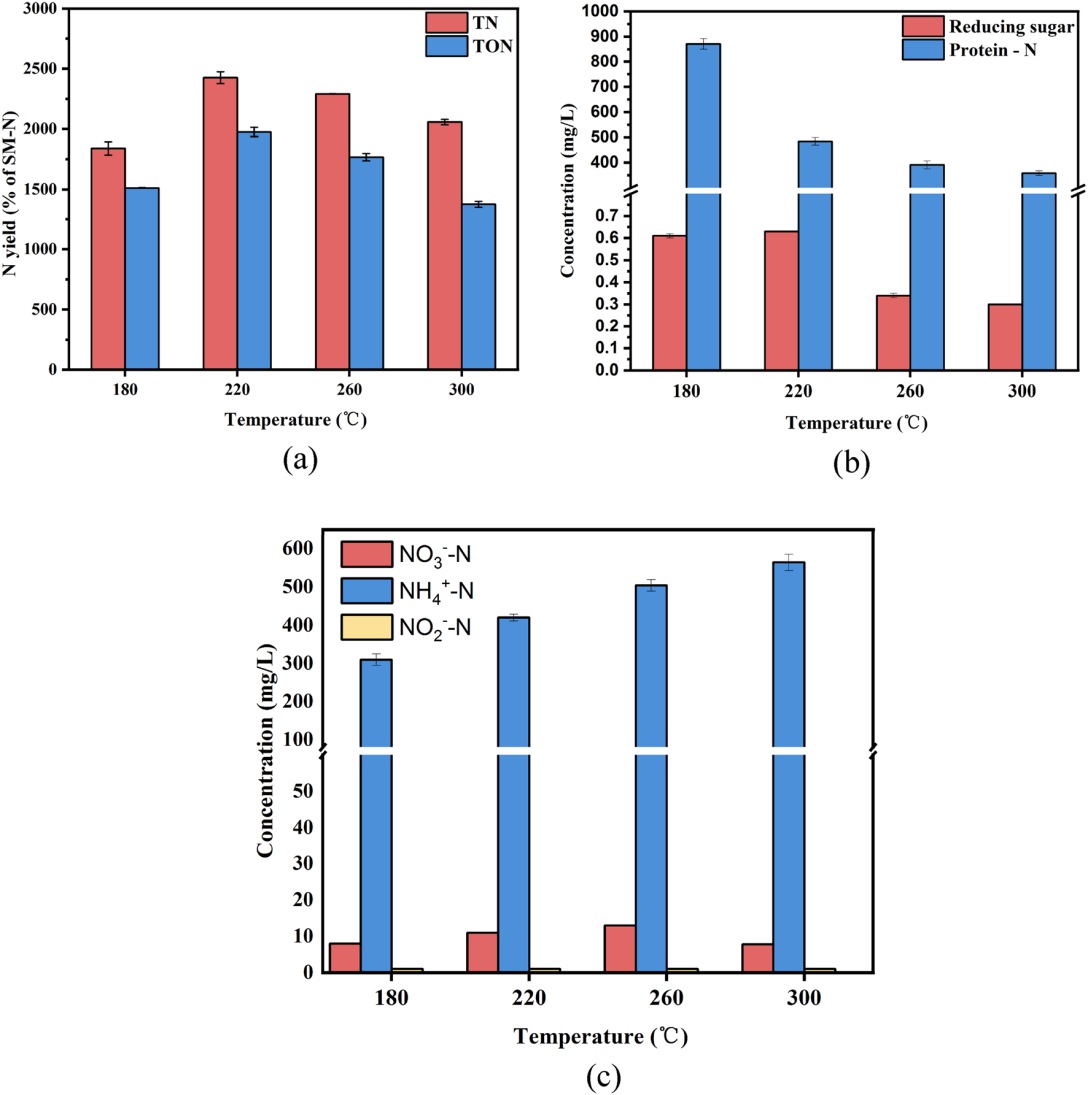
Effect of temperature on the concentration change of N in liquid phase products: (**a**) TN and TON, (**b**) Protein-N and reducing sugar, (**c**) NH_4_^+^–N, NO_3_^−^–N and NO_2_^−^–N.

It is well-known that TN consists of ON (organic nitrogen) and ION (inorganic nitrogen, including NH_4_^+^–N, NO_3_^−^–N and NO_2_^−^–N)^32^. Of these, we clearly observed that Protein-N, NH_4_^+^–N and NO_3_^−^–N, three N containing species existed in the liquid phase products, whereas the content of NO_2_^−^–N and was much lower than the first three, according to previous studies^33^. As stated by Ekpo et al. and Liu et al.^34,35^, the temperature play a vital role in nitrogen solubilization. Figure 2a shows that TN concentration underwent an obvious two-stage throughout the thermochemical liquefaction process. In the first stage (180–220 °C), the concentration of TN increased dramatically from 1840.7 to 2426.3 mg/L, which may be attributed to protein-N and inorganic-N conversion into NH_4_^+^–N, NO_3_^−^–N, and NO_2_^−^–N by hydrolysis. In general, the free state of amide nitrogen was formed via the break of peptide bonds from protein-N, followed by deamination and ring opening reaction to form NH_4_^+^–N. Lower reaction temperature (below 220 °C) facilitated the deamination of labile amides, while a violent condition was required for the ring opening reaction^36^. Further, the significant increase in ON in this stage indicated that the degradation of protein-N (form dissoluble ON) prevailed over the hydrolysis of amide-N (form NH_4_^+^–N). The fact described above is in line with the previous research^37^, and from Fig. 2b, the sustained decrease of protein nitrogen concentration could also support it. During the second stage (220–300 °C), the concentration of TN reduced gradually from 2426.3 to 2057.8 mg/L. The highest TON concentration was also observed at 220 °C (1975.2 mg/L), and it exhibited the same decreasing tendency with TN concentration as elevated temperature. Due to the accumulation of protein with the thermochemical liquefaction process and the higher temperature, the protein hydrolysis rate was faster than the cellulose degradation rate, resulting in a constant reduction of TON concentration. The levels of NH_4_^+^–N, NO_3_^−^–N, and NO_2_^−^–N are shown in Fig. 2c. The slight increase in NH_4_^+^–N (from 310.5 to 564.9 mg/L), the main inorganic nitrogen, could be ascribed to the break of pyridine nitrogen and stable amide nitrogen in biochar, bio-oil, and liquid phase products. We did not discuss the concentration change of NO_3_^−^–N and NO_2_^−^–N because of their low content after the thermochemical liquefaction process (less than 1% of TN-N). Further, except for the nitrogen in liquid phase products, a plunge of reducing sugar was detected. This was due to the degradation of the non-fibrous carbohydrates of SM. The reducing sugar concentration peak was at 220 °C (0.63 mg/L), decreasing dramatically to 0.33 mg/L as the temperature rose to 260 °C. This suggests that the reducing sugar was transformed to organic acids and other intermediate materials (CO_2_) by thermochemical liquefaction^38^.

### Effect of temperature on nitrogen evolution in biochar during thermochemical liquefaction

**Figure 3:**
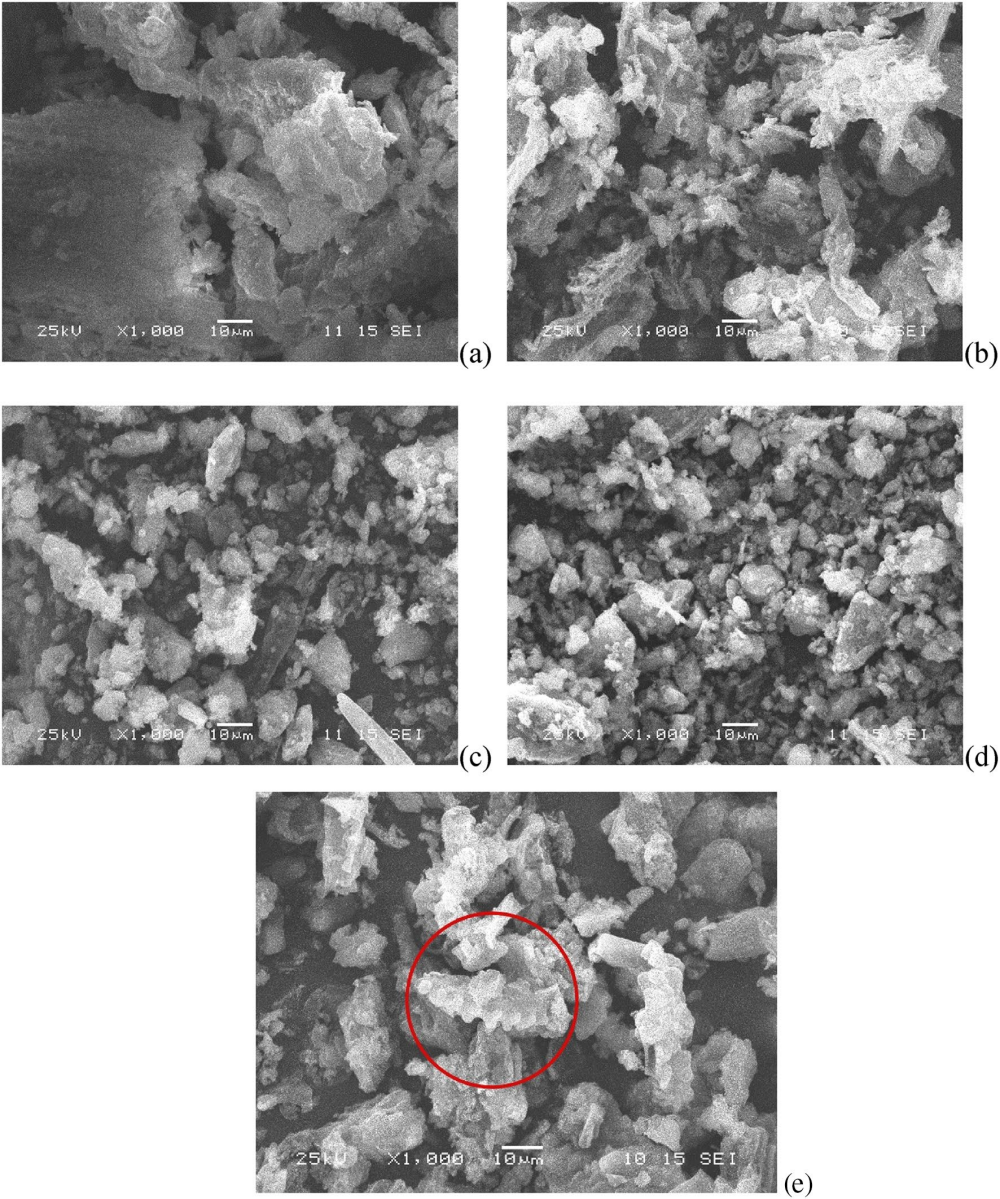
SEM images of SM and biochar surfaces from various thermochemical liquefaction temperatures with ×1000 magnification: (**a**) SM, (**b**) 180 °C, (**c**) 220 °C, (**d**) 260 °C, and (**e**) 300 °C.

The nitrogen-rich biochar from thermochemical liquefaction could be used in heavy metal adsorbents, compost additive and other areas. The SEM images were helpful in studyding the morphological changes from SM to biochar under different temperatures. Compared with biochars (Fig. 3b–e), SM (Fig. 3a) had a relatively smooth surface and dense matrix. Further, part of the biochar surface was still dense. This phenomenon suggested that thermochemical liquefaction did not completely destroy the molecular chains of biochar, which might be due to the adoption of ethanol as a solvent^39^. On the surface of the biochar, a flocculent structure composed of smaller particles was observed. These smaller particles mostly came from the degradation of cellulose whereas SM contained less cellulose than other fecal biomasses. As the reaction temperature continued to rise (220–300 °C), the size and quantity of the cross-linking structures increased more obviously than did that from 180 to 220 °C. This indicated that increasing temperature promoted polymerization and condensation^40^. Meanwhile, the nitrogen content in biochar showed little fluctuation at this range, which might be attributed to the generation of basic particles. It was noteworthy that a serrated rod-shaped structure of biochar was observed, as shown in Fig. 3e, where quaternary nitrogen content showed a sharp increase. These nitrogen changes may affect the evolutionary process of the microscopic structure of biochars. In the study by Wang et al.^41^, the biochar from spirulina biomass exhibited a similar microscopic structure. Kruse et al.^42^ showed that combining wood biomass with amino acid cysteine could produce biochar with “berry” structure. The biochar generated from a hydroxymethylfurfural/cysteine combination validated this performance. Therefore, it could be inferred that the pathways of biochar formation from N-containing biomass (such as protein and algae) with carbohydrates as additives differed from those of single carbohydrates as the feedstock.

**Figure 4:**
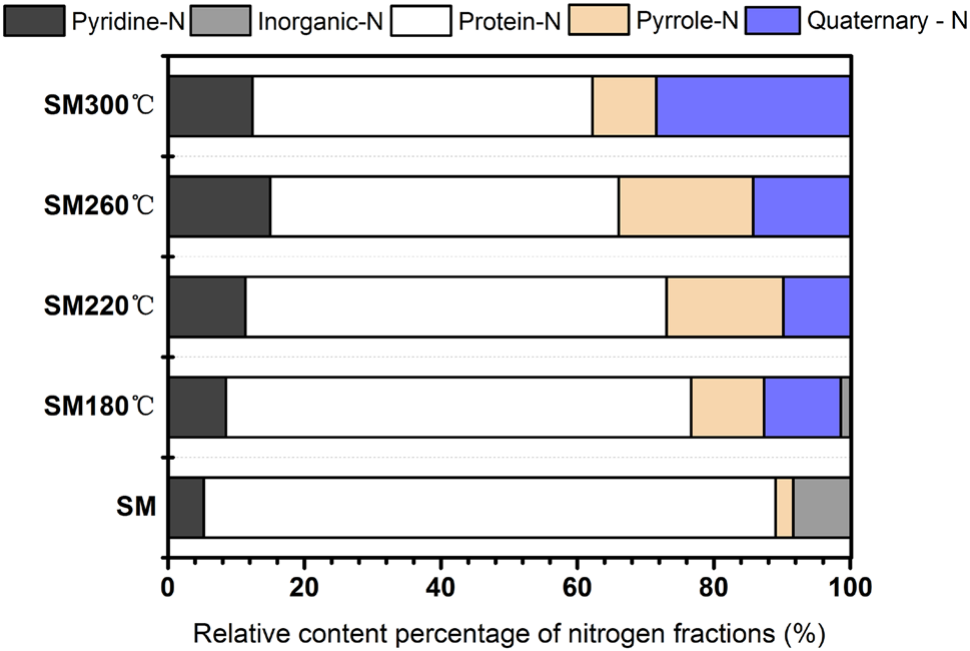
Nitrogen species distribution in SM and biochar.

XPS was used to better understand the evolution of existing nitrogen forms in biochar during thermochemical liquefaction at various temperatures. The nitrogen species distribution in SM and biochar is shown in Fig. 4 and Table B (Supplementary). The nitrogen 1S spectra are given in Fig. A (Supplementary). There were a few peaks of the nitrogen spectra of biochar obtained from SM thermochemical liquefaction: inorganic-N (403 ± 0.2 eV, may include ammonia-N or nitrates-N/nitrites-N), quaternary-N (401.3 ± 0.2 eV), pyrrole-N (400.2 ± 0.2 eV), protein-N (399.8 ± 0.2 eV), and pyridine-N (398.8 ± 0.2 eV). As Fig. Aa illustrates, four peaks were involved in the SM, including pyridine-N, protein-N, pyrrole-N, and inorganic-N. The contents of these functionalities (pyridine, protein, pyrrole, inorganic) were 5.25%, 83.78%, 2.62%, and 8.55%, respectively. This suggests that protein nitrogen and pyridine nitrogen were the major functionalities in the raw SM. After thermochemical liquefaction treatment, there was a significant difference between biochar and feedstock. Quaternary nitrogen was first generated at 180 °C (11.28%), and protein nitrogen was reduced to 68.17%. The reduction of protein nitrogen content indicated that a fraction of protein nitrogen was deaminated during thermochemical liquefaction, and nitrogen was transferred to bio-oil and liquid-phase products, which was also in line with the result of Fig. 1^21^. In particular, the content of reducing sugar obtained from the hydrolysis of cellulose was relatively high at this point. It can be inferred that the presence of quaternary nitrogen in biochar at 180 °C (Fig. 4) was due to active Maillard reactions between reducing sugar and protein. The inorganic nitrogen content of biochar had a remarkable decrease compared to that of the SM, and even disappeared at 260 °C and 300 °C. This was reasonable to believe that the thermal stability of inorganic-N was weakened with elevated temperature, and the hydrolysis and dissolution gradually converted most inorganic nitrogen in the biochar into NH_4_^+^–N and NH_3_–N^43^. When the temperature increased from 220 to 260 °C (Fig. 4), the content of protein-N continued to decrease from 61.70 to 51.09%, coupled with pyridine nitrogen and pyrrole nitrogen increased by 3.63% and 2.57%, respectively. The content of quaternary nitrogen was lower than that of pyridine nitrogen and pyrrole nitrogen, suggesting that amino acids tended to be transformed to the latter in this temperature range. These results were similar to those of Wang et al.^44^, who used food waste as a nitrogen resource for hydrothermal carbonization. As previously observed the presence of glucose (as a kind of reducing sugar) enhanced the aromatization of nitrogen^45^. Figure 2b clearly shows that the reducing sugar content underwent a sharp decrease with rising temperature and the Maillard reactions were delayed due to a lack of reactants. Meanwhile, the quaternary nitrogen in biochar increased steadily, accompanied by a decrease in pyridine nitrogen and pyrrole nitrogen. These results demonstrated that the rising temperature promoted pyridine-N and pyrrole-N polymerized to form a more stabilized nitrogen formation (such as quaternary nitrogen) by ring condensation reactions^46^. In conclusion, XPS and the concentration of reducing sugar indicated that the reducing sugar in SM favored fixing nitrogen in the biochar. Moreover, high temperatures favored the incorporation of nitrogen into aromatic rings.

FTIR was conducted to understand the surface functional group of biochar under different reaction temperatures during thermochemical liquefaction (Fig. B, Supplementary). The absorption peaks observed about 3700–3200 cm^−1^ in biochar corresponded to the O–H and N–H, and can be attributed to the action of hydroxyl groups and amino groups, respectively. These two peaks were found to be weak in biochar 300 because the high temperature promoted the thermal dehydration reaction. The peaks of the spectra from all biochar between 2900 and 2800 cm^−1^ was attributed to the aliphatic C–H stretching vibration, reflecting the generation of aliphatic structures after thermochemical liquefaction^22^. The bands about 1650 cm^−1^ was assigned to C=O stretching in the protein, which was in line with the XPS results. The peak appearing between 1460 and 1480 cm^−1^ in all biochar samples corresponded to the C=C stretching in aromatic groups, and was slightly increased as temperature increased^47^. The characteristic peaks around 1250 cm^−1^ and 1130 cm^−1^ in biochar were ascribed to C–N and C–O (–CO–NH) group, which was increased in biochar 260 and biochar 300. This indicated that the peptide bond was formed by dehydration and condensation of amino acids. There was also a weak peak at about 1690 cm^−1^. The use of ethanol may lead to the generation of a carboxyl, carbonyl, or ester group in biochar, suggesting the presence of oxygen-containing functional groups on the surface of biochar^48^.

### Effect of temperature on the nitrogen content of bio-oil during thermochemical liquefaction

**Figure 5:**
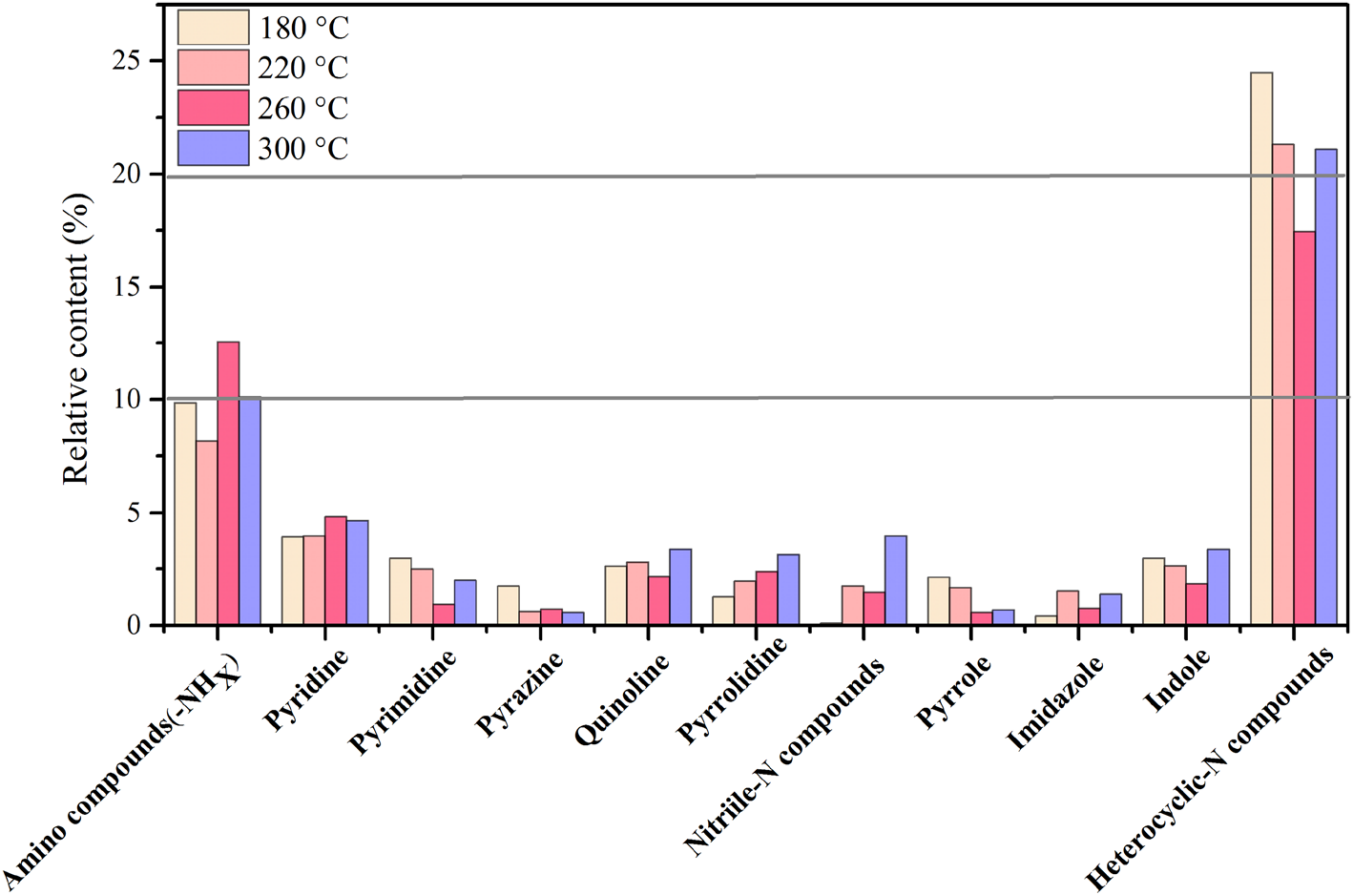
The major N-containing compounds species distribution in bio-oil.

The relative content of the nitrogen-containing compounds in bio-oil from thermochemical liquefaction was studied by GC–MS to determine the nitrogen fate. In the oil refining industry, the presence of steric hindrance in high molecular nitrogen-containing compounds prevents them from accessing the surface of the catalyst, which increases the difficulty of hydrodenitrogenation. Figure 5 shows that nitrogenous compounds in bio-oil were greatly unstable to change because of the effect of rising temperature. As the temperature climbed from 180 to 260 °C, the amine compounds (–NHx) fractions increased gradually and reached the highest content of 12.55%. A similar trend was found for pyrolysis, which uses sewage sludge as raw material^49^. This finding indicates that the emerging reaction temperature could promote the conversion of protein nitrogen into amine compounds (–NHx)^50^. Notably, the content of pyridine also increased from 3.93 to 4.83%, showing the same tendency as the content of amine compounds (–NHx). It was reasonable to infer that the amine compounds (–NHx) were condensed into intermediate products and generated pyridine nitrogen compounds based on the Diels–Alder reaction^51^. By comparison to other studies that used water as a solvent, this phenomenon suggested that ethanol could favor the accumulation of nitrogen in bio-oil and the Diels–Alder reaction. Further, as the representative of heterocyclic nitrogen compounds, pyrrole and pyrazine derived from the cracking reaction of amino acids showed the highest value at 180 °C (2.13% and 1.74%, respectively). However, as the temperature increased, the relative fractions of pyrazine-N compounds obtained from the reaction between inorganic nitrogen and sugar cyclic oxygenates were gradually reduced. The results confirmed that a higher thermochemical liquefaction temperature promoted the transformation of the sugar cyclic oxygenates into other forms of nitrogen^50^. In the thermochemical liquefaction process, the presence of reducing sugar was use to the degradation of non-fibrous carbohydrates of SM as was mentioned before^21^. Amino acids interact with reducing sugar produced by the hydrolysis of carbohydrates to form heterocyclic nitrogen compounds, such as indole, quinoline, pyrrolidine, and imidazole. These heterocyclic nitrogen compounds are also produced by the cyclization of amine nitrogen intermediates derived from the thermal cracking of stable proteins^52^. As the reaction temperature increased, the amount of these compounds showed an inconsistent increase, a trend that was also reported in previous research^53^. This indicates that high temperatures favor the further cyclization and condensation of heterocyclic nitrogen and that these intermediate products play an important role in the nitrogen transformation of the thermochemical liquefaction process. Furthermore, as Fig. 5 illustrates, there was an obvious increase in nitrile nitrogen compounds when the temperature was above 180 °C, and its relative fractions reached the peak at 300 °C (3.97%). In general, nitrile nitrogen compounds were mainly prepared by nucleophilic substitution^54^. However, in the thermochemical liquefaction process, it is derived from dehydration reactions of amides intermediates and cellulose derivatives at high temperature^55^.

These findings also show that ethanol can be effectively used as a solvent to extract N-containing compounds in bio-oil during the thermochemical liquefaction process. The major products in bio-oil were produced from the direct cracking of protein and the polycondensation of the N-heterocyclic compound, which was closely related to reaction temperature.

### Nitrogen conversion and possible reaction schematics during the SM thermochemical liquefaction

**Figure 6:**
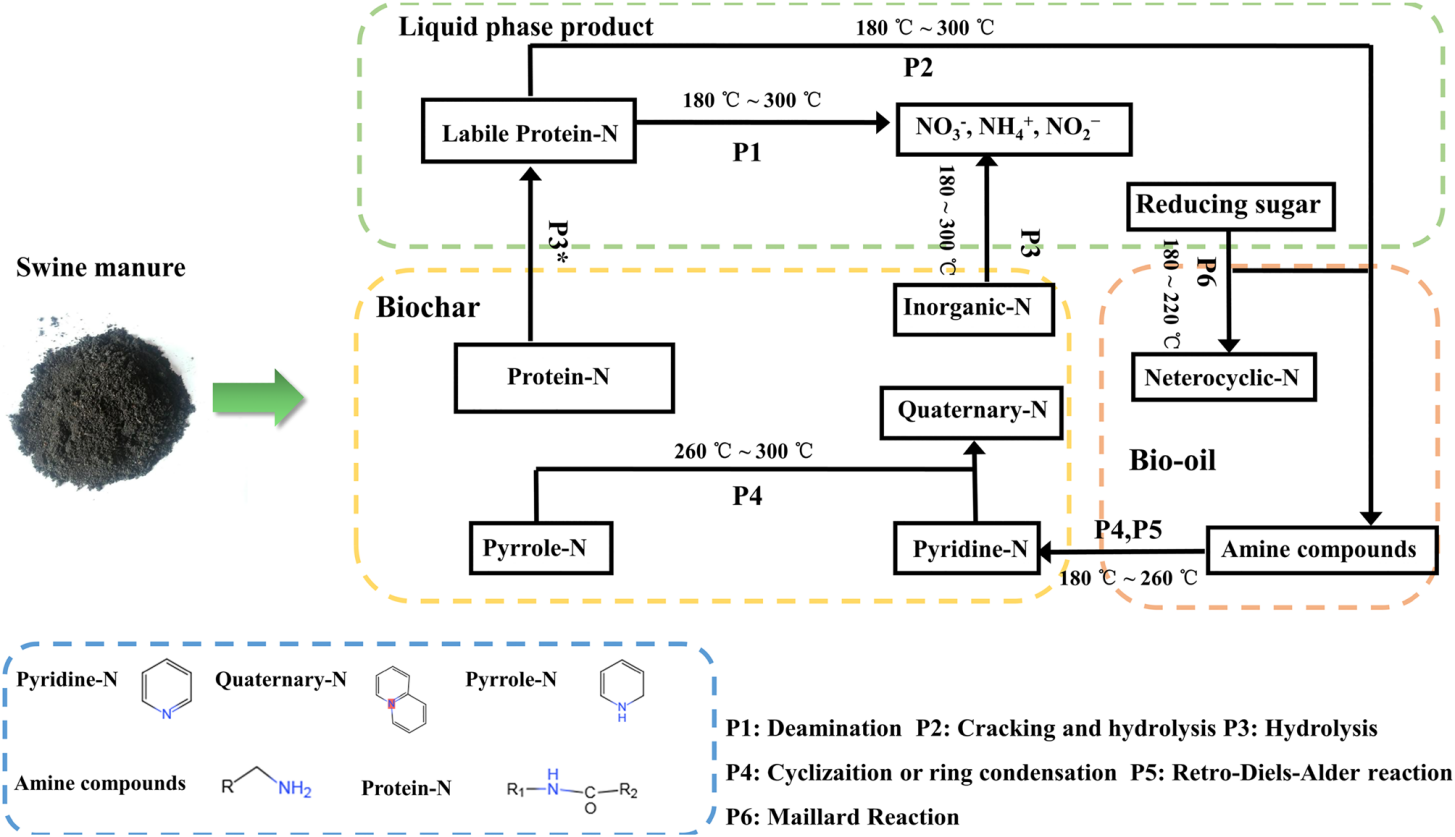
Possible nitrogen conversion pathways of SM during thermochemical liquefaction.

Compared with other livestock manures, SM has a higher nitrogen content and protein-N^56^. Thermochemical liquefaction of SM could lessen the burden on the environment as well as endow the energy value for SM. Based on the above analysis, possible nitrogen conversion pathways of SM during thermochemical are proposed in Fig. 6. XPS spectra indicated that nitrogen in biochar was comprised of pyridine nitrogen, protein nitrogen, pyrrole nitrogen, inorganic nitrogen, and quaternary nitrogen. The inorganic nitrogen in the SM and biochar was hydrolyzed to the liquid phase fraction in the form of NH_4_^+^–N, NO_3_^−^–N, and NO_2_^−^–N (P3), resulting in the subsequent increase of inorganic nitrogen content in the liquid phase fraction. The rising temperature with the thermochemical liquefaction process promoted the cyclic polycondensation of pyridine nitrogen and pyrrole nitrogen to form a ring structure on the polymer molecular chain via the Diels–Alder reaction, which led to an increase in quaternary nitrogen (P4 and P5). Labile protein nitrogen in the liquid phase fraction was partially converted into amine compounds by cracking the peptide bond and hydrolysis (P2). Another portion of labile protein nitrogen decomposed via deamination and accumulated NH_4_^+^–N (P1). Maillard reactions were the main approach to produce some heterocyclic nitrogen compounds in this study; typical heterocyclic nitrogen compounds, such as pyrrole, pyrazine, pyridine, etc., were detected in bio-oil. These heterocyclic nitrogen compounds were also formed by cracking and hydrolyzing labile protein nitrogen (P2 and P6). The relatively high temperatures (above 220 °C) and decomposition of carbohydrates would weaken the Maillard reaction; however, the polyfuran/aromatic network were transformed to labile aromatic structures in biochar and formed building blocks (carbon precursors), which promoted nitrogen combination^57^. In other words, the cyclization or condensation of amine compounds in bio-oil from protein led to an enhancement of the pyridine nitrogen proportion in the thermochemical biochar at 180–260 °C (P4 and P5), suggesting that the deamination of protein nitrogen was enhanced at this point. Thus, it can be inferred that temperature and reaction substances were the main factors impacting the formation of heterocyclic nitrogen compounds.

### Economic analysis and energy assumptions

The techno-economic analysis of transportation fuel production via thermochemical liquefaction is shown in Table 2. Their minimum selling price was regarded as an economic indicator to evaluate economic feasibility. Significant variations in the minimum selling price were attributed to feedstock type, plant size, and co-product utilization. The price ranged from 0.76 to 2.06 **$**/kg. Supported by Fischer–Tropsch synthesis, the economic analysis of biofuels from thermochemical liquefaction has led to a breakeven cost of diesel of 1.30 **$**/L that could be attained by the biomass feedstock. Alherbawl et al. found that drop-in bio-oil from animal manure thermochemical liquefaction could be sold for 0.87 **$**/kg and an emissions reduction of 7% was achieved compared to traditional gasoline^58^. The price of swine manure and other consumptions are summarized in Table C (Supplementary). Since our study is focused on speciation and transformation of nitrogen during thermochemical liquefaction, it is assumed that our experimental process is conducted in an ideal state and the benefit of dealing with swine manure could offset the cost of transportation and others. The cost of our experiment is estimated to be about 0.35 **$**/kg (laboratory level, the cost of swine manure was set to zero). To accomplish the commercial applications of thermochemical liquefaction of swine manure, this technology require further improvement in reducing high production cost and increasing yield of bio-oil.

**Table 2:** Techno-economic analysis of transportation fuel production via thermochemical liquefaction.

Feedstock	Plant size	End products	Minimum selling price	References
Aspen/residual wood	1000 ton/day	Gasoline-equivalent/Jet fuel	1.06–1.48 **$**/kg	^62,63^
Lignocellulose residue	1500 ton/day	Gasoline-equivalent	1.14 **$**/kg	^64^
Forestry residues	3000 ton/year	Gasoline-equivalent	1.06–1.16 **$**/kg	^65^
Algae	Algae productivity: 34–57 g/m^2^/day	Biofuel	0.76 **$**/kg	^66^
Wastewater-based algae	Algae productivity: 25 g/m^2^/day	Diesel	2.06 **$**/kg	^67^

The energy assumptions was listed as follows: (1) Before thermochemical liquefaction, the chemical energy contained in the swine manure is not considered. (2) Compared to the heats required in our process, heat loss through the reactor walls was negligible. (3) According to previous research^59^, the specific heats of the swine manure are equal or less than that of water. In other words, the required energy to heat pure water to setting temperature is more than that of swine manure. (4) The change in actual value are lower than the internal energy, the actual energy required to heat the other solutions (ethanol) are lower than that of water. Therefore, the energy input used in thermochemical liquefaction is conservative.

The maximum set temperature of thermochemical liquefaction is 300 °C and the pressure is 0.1 Mpa. From V.Babu^60^, the internal energy of water at 300 °C and 27 °C is 1350 kJ/kg and 93 kJ/kg, respectively. The required energy is: 1350–93 kJ/kg = 1257 kJ/kg. Heat is the main energy source of thermochemical liquefaction and could be supplied by combustion of natural gas, which has an industrial cost of 0.447 **$**/m^3^ in China^61^. Perfect combustion of a cubic meter of natural gas could produce 38 MJ (10.6 kWh), and the cost of energy is calculated to be 0.014 **$**/kg.

## Conclusion

This study investigated the fate of the nitrogen in SM during thermochemical liquefaction different temperatures for the first time, provided an understanding of nitrogen conversion mechanism. Thermochemical liquefaction using ethanol as a solvent could improve bio-oil quality and transform nitrogen into more stable forms by hydrolysis, polymerization, and deamination. The majority of N in SM was converted into bio-char, bio-oil, and liquid phase products; a large amount of bio-char-N migrated to liquid-N and bio-oil-N as the reaction temperature increased. A large proportion of pyridine nitrogen was transformed to protein nitrogen at 260–300 °C in biochar via the Diels–Alder reaction. As for bio-oil, amine compounds and heterocyclic nitrogen derived from the hydrolysis of proteins and the Maillard reaction were dominant products. During thermochemical liquefaction, the Maillard reaction occurred at 180–220 °C and high temperature would inhibit it. Notably, nitrile nitrogen was derived from dehydration reactions of amide intermediates and cellulose derivatives rather than from nucleophilic substitution in this study. At 260–300 °C, the cyclization and condensation of heterocyclic nitrogen were enhanced, which caused the increase of indole, quinoline, pyrrolidine, and imidazole in bio-oil. This study proposed a reaction pathway for nitrogen that played a vital part in improving bio-oil and biochar quality from SM. However, further investigations are required to discover rawer materials to produce bio-oil and biochar from thermochemical liquefaction. The characteristics of bioproducts from the thermochemical liquefaction process need to be comprehensively explored in the future.

## Methods

### Materials

SM was collected from the local pig farm of Hunan. Before the thermochemical liquefaction process, the fresh feedstock was dried at 100 °C for 24 h (19.2 kWh), pulverized and screened through 80 mesh. The elemental composition of bio-oil and biochar was analyzed using a Flash EA-1112 Elemental Analyzer (USA). The proximate and ultimate analysis of SM, as well as the higher heating value, are listed in Table 3 and performed with previous studies^35^. The reagents used in the liquefaction experiments were analytical grade (anhydrous ethanol, ethyl acetate, and deionized water). All experiments were performed in triplicate, and the mean value was reported.

**Table 3:** Characteristics of SM.

Proximate analysis (wt%, db^a^) Physical and chemical characteristics							Ultimate analysis (wt%, daf^b^)					HHV (MJ/kg)
VM	MC	Ash	FC	pH	Ammonium	Nitrate	C	H	O^d^	N	S	
58.40	75.90^c^	22.30	8.40	7.89	0.39 g/kg	15 mg/kg	35.23	5.26	56.4	2.68	0.41	13.24

^a^db, dry basis.

^b^daf, dry and ash.

^c^Original moisture content.

^d^Calculated by difference.

### Experimental apparatus and procedure

Thermochemical liquefaction of SM was conducted in a 500 mL autoclave reactor (GSHA-0.5, China) outfitted with a magnetic stirring mechanism, electronic furnace, thermometer, and pressure gauge. In each trial, 10 g of SM sample and 100 mL of anhydrous ethanol were placed in the reactor. The starting temperature and pressure in the reactor was 27.3 °C and 0.1 Mpa. The sealed reactor was heated to 180–300 °C (in increments of 40 °C, approximately 5 °C/min) via an external electric furnace and maintained for 25 min with stirring using a magnetic stirrer speed of 70 rpm. Once the liquefaction process was finished, the reactor was cooled down to ambient temperature.

**Figure 7:**
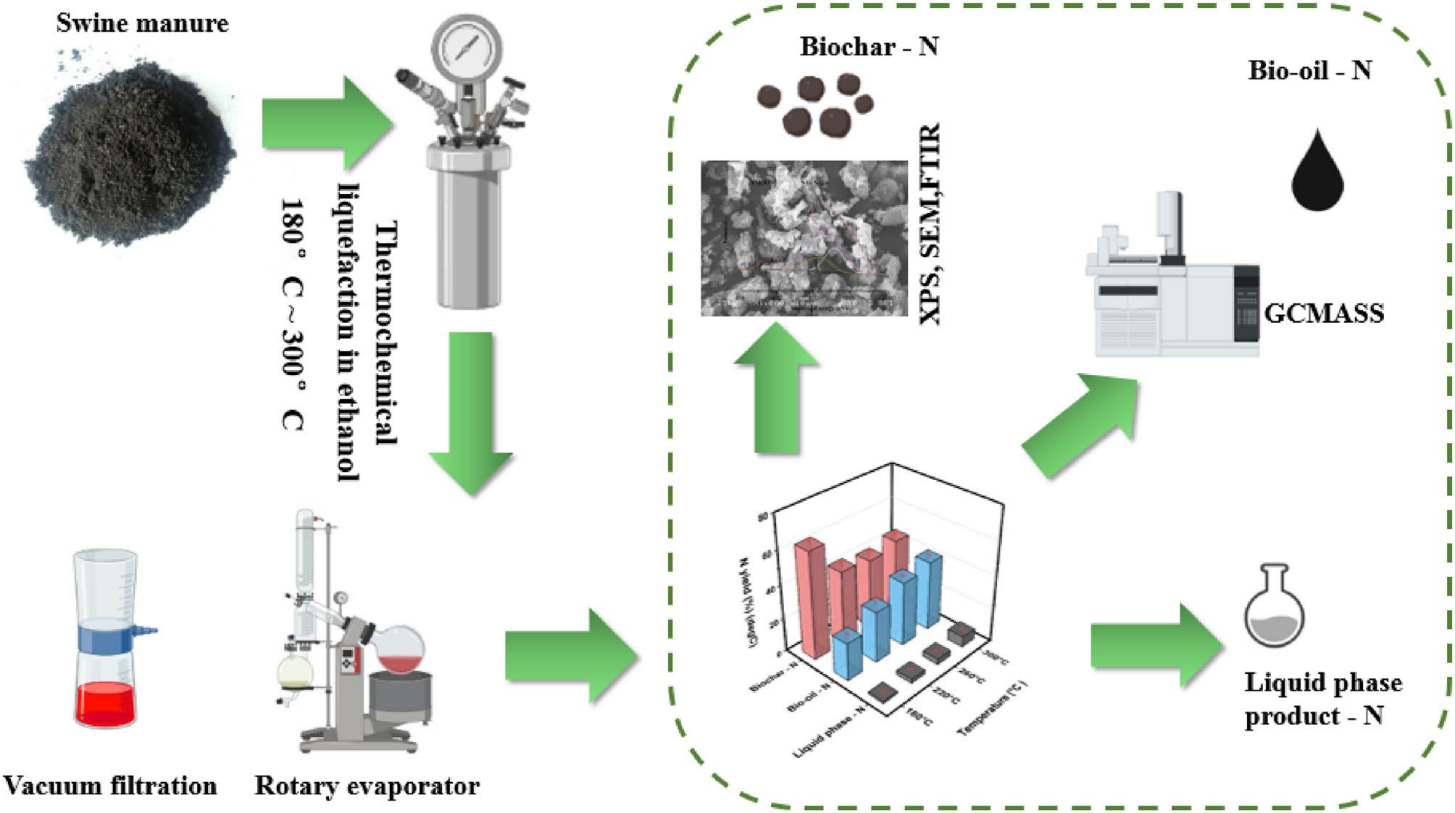
The whole experiment procedures.

The solid–liquid mixture was transferred to a vacuum filtration apparatus to separate from each other. The solid phase product was dried in an oven at 100 °C for 24 h, which is referred to as biochar. The biochar was transferred to a beaker and weighed for further testing. A rotary evaporator (RE-2000B, China) was used to remove the organic solvent (ethanol, ethyl acetate) and separate liquid and oil phase products (referred to as bio-oil). The seperated bio-oil and liquid phase products were stored in sealed glass bottle for further study. All trials were repeated three times. The whole process is shown in Fig. 7.

### Analysis of liquid phase product

In this study, the total nitrogen (TN) could be estimated as follows: TN = TON (total organic nitrogen) + (NH_4_^+^–N, NO_3_^−^–N). The Kjeldahl content in the liquid phase product could represent the total nitrogen, which could be detected by following GB11891-1989 (China). The ammonium and nitrate were measured on the basis of previous research (colorimetric method and salicylic acid nitrification method)^20,68^. Protein-N was estimated as the following formula:

1 $$ {\text{Protein{-}N }} = {\text{ PC}}/{6}.{25,} $$

where PC was the protein concentration and 6.25 was the conversion fraction.

The bicinchoninic acid method (BCA), an extremely sensitive and selective detection reagent, eliminated the soluble protein in the liquid phase product using a protein assay kit^69^. DNS method was used to determined the content of reducing sugar in liquid phase products. The DNS method was used to determine the content of reducing sugar in the liquid phase products. The preparation method for the DNS reagent was as follows: 6.3 g 3,5-dinitrosalicylic acid and 262 mL 2 mol/L sodium hydroxide were added to 500 mL hot water solution containing 182 g sodium potassium tartrate, and 5 g phenol and sodium sulfite were added. The mixture was stirred until it was completely dissolved. After cooling, water was added to a constant volume of 1000 mL to prepare the DNS reagent, which was stored in a brown bottle for one week.

### Analysis of bio-oil products

Bio-oil was extracted by ethyl acetate and underwent rotary evaporation. The GC–MS technique was used to identify the N-containing species in bio-oil (Agilent 7890B 5977 A GC/MSD instrument with a built-in HP-5 ms capillary column, 5% biphenyl + 95% dimethylpolysiloxane, 30 m × 0.25 mm × 0.25 μm), detailed parameters can be found in previous paper^35^. The N-containing compounds in the bio-oil corresponding to the main peak were compared with NIST mass spectral data library. The higher heating value (HHV) of bio-oil and biochar was calculated according to Dulong’s function (Supplementary)^35^. The bio-oil-N was determined for TON by subtracting the protein-N.

### Analysis of biochar products

The species and proportion of N in SM and biochar were evaluated using X-ray photoelectron spectroscopy, (XPS, Thermo ESCALAB 250XI, USA). The identical pass energy and energy step size were 20 eV and 0.1 eV, respectively, employing an Al K X-ray source. The scanning of analysis was set at 8–10 times on solid surface to ensure sufficient quality. The functional groups of SM and biochar obtained at different reaction temperature were determined by Fourier transform infrared spectroscopy, (FTIR, Nicolet iS5 spectrometer, Thermopylae Nicolet, America). Before the determination 1 mg samples were mixed with 100 mg of KBr samples and pressed into slices, then transferred in the 4000–400 cm^−1^ region with 100 scans. The nitrogen mass in the bio-char was calculated using the following formula:

2 $$ {\text{N}}_{{{\text{biochar}}}} = {\text{ TM}}_{{{\text{biochar}}}} \times {\text{ T}}_{{{\text{nitrogen}}}} , $$

where TM_biochar_ was the total mass of biochar and T_nitrogen_ was the mass ratio of nitrogen. Further, the surface morphology of biochar was measured by a scanning electron microscope with WD (working distance) as 8 mm (JSM-6380LV, Japan).

## Supplementary Information

Supplementary Information.

## Data Availability

All data generated or analysed during this study are included in this published article and its supplementary information files. (We confirmed the data used to support the findings of this study are available from the corresponding author upon request).
