# Supplementary material for: Speciation and transformation of nitrogen for swine manure thermochemical liquefaction
Source: Sci Rep. 2022 Jul 14;12:12056. doi: 10.1038/s41598-022-16101-w (PMC9283412; doi:10.1038/s41598-022-16101-w)
Supplement: Supplementary file 1 — Supplementary Information. [file 41598_2022_16101_MOESM1_ESM.docx]

# Supplementary Information

Dulong’s function:

HHV = 0.3383C + 1.442 (H–O/8)

where HHV, the short for higher heating value; C, the mass percentage of carbon; H, the mass percentage of hydrogen and O, the percentage of oxygen.

**Table A**

| Sample | N (wt%) | C (wt%) | H (wt%) | S (wt%) | O ^c^ (wt%) | HHV (MJ/kg) |
| --- | --- | --- | --- | --- | --- | --- |
| BO ^a^180 ℃ | 3.50 ± 0.02 | 66.55 ± 0.05 | 8.43 ± 0.22 | -^b^ | 21.52 ± 0.32 | 30.68 |
| BO220 ℃ | 3.58 ± 0.05 | 74.66 ± 0.06 | 9.05 ± 0.45 | -^b^ | 12.31 ± 0.03 | 35.94 |
| BO260 ℃ | 3.39 ± 0.04 | 73.18 ± 0.12 | 11.24 ± 0.07 | -^b^ | 12.19 ± 0.07 | 38.58 |
| BO300 ℃ | 4.06 ± 0.04 | 75.91 ± 1.08 | 11.13 ± 0.21 | -^b^ | 10.70 ± 3.71 | 39.62 |

Elemental analysis and HHV of bio-oil and biochar.

^a^ BO, bio-oil

^b^ -, undetectable

^c^ Oxygen content was calculated by difference method: O% = 100 − (C% + H% + N% + S%)

**Table B**

XPS deconvolution results of SM and biochar.

| Sample entry | Normalized relative intensities of nitrogen functionalities (%) | | | | |
| --- | --- | --- | --- | --- | --- |
|  | 398.8 eV | 399.8 eV | 400.2 eV | 401.4 eV | 402.9 eV |
|  | Pyridine-N | Protein-N | Pyrrole-N | Quaternary-N | Inorganic-N |
| SM | 5.25 | 83.78 | 2.62 | 0 | 8.55 |
| SM180℃ | 8.51 | 68.17 | 10.66 | 11.28 | 1.38 |
| SM220℃ | 11.36 | 61.70 | 17.12 | 10.03 | 0.76 |
| SM260℃ | 14.99 | 51.09 | 19.69 | 14.21 | 0 |
| SM300℃ | 12.39 | 49.83 | 9.34 | 28.44 | 0 |

**Table C.** Price of swine manure, chemicals and utilities (in 2022 U.S.dollars)

| Consumptions | Value |
| --- | --- |
| Swine manure | 11.98 $ metric ton (Minimum market price) |
| Electricity | 0.09 $/kWh |
| Cooling water | 0.367 $ metric ton |
| Chemicals | 11.96 $ litre |

**
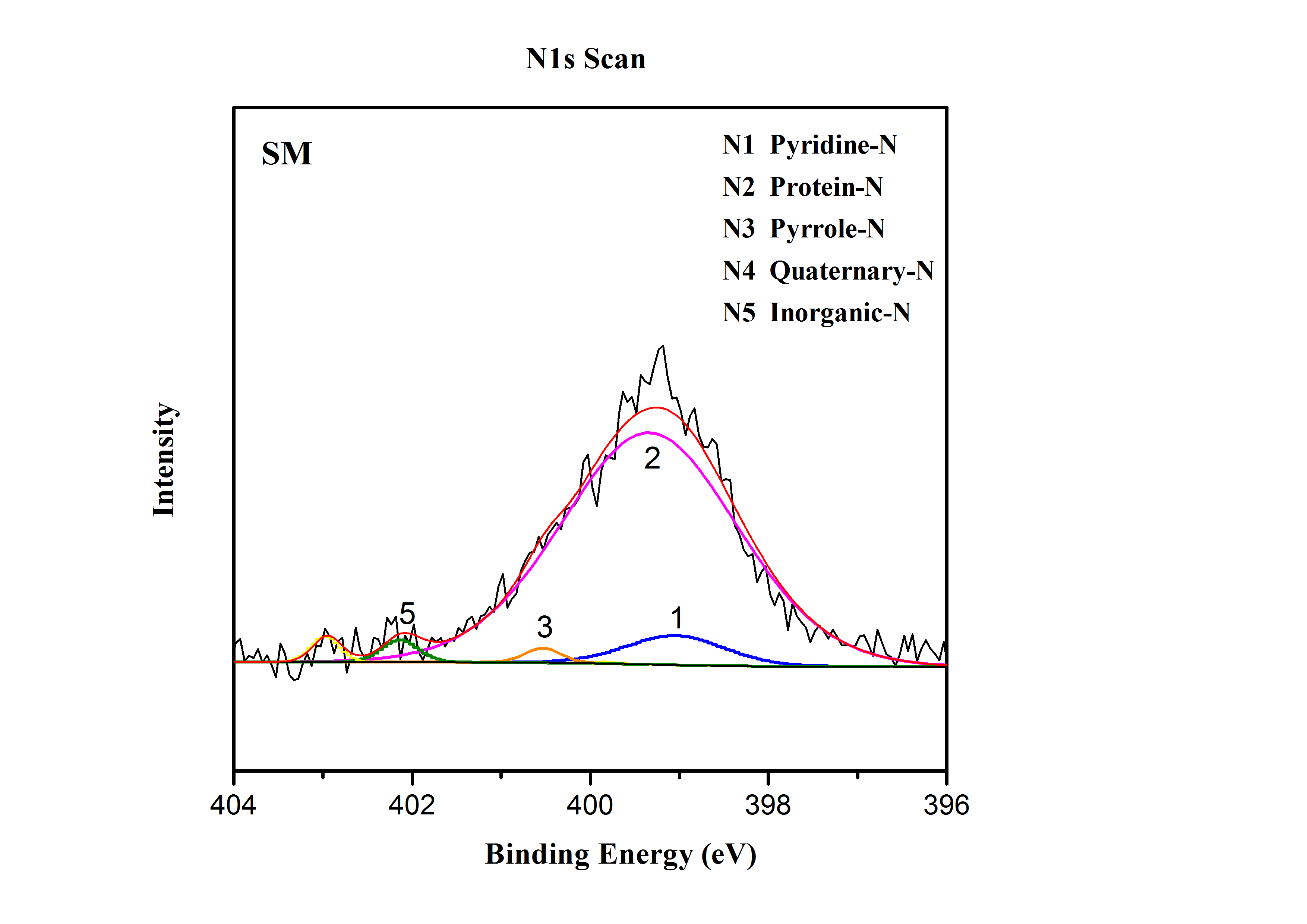
**

(a)

**
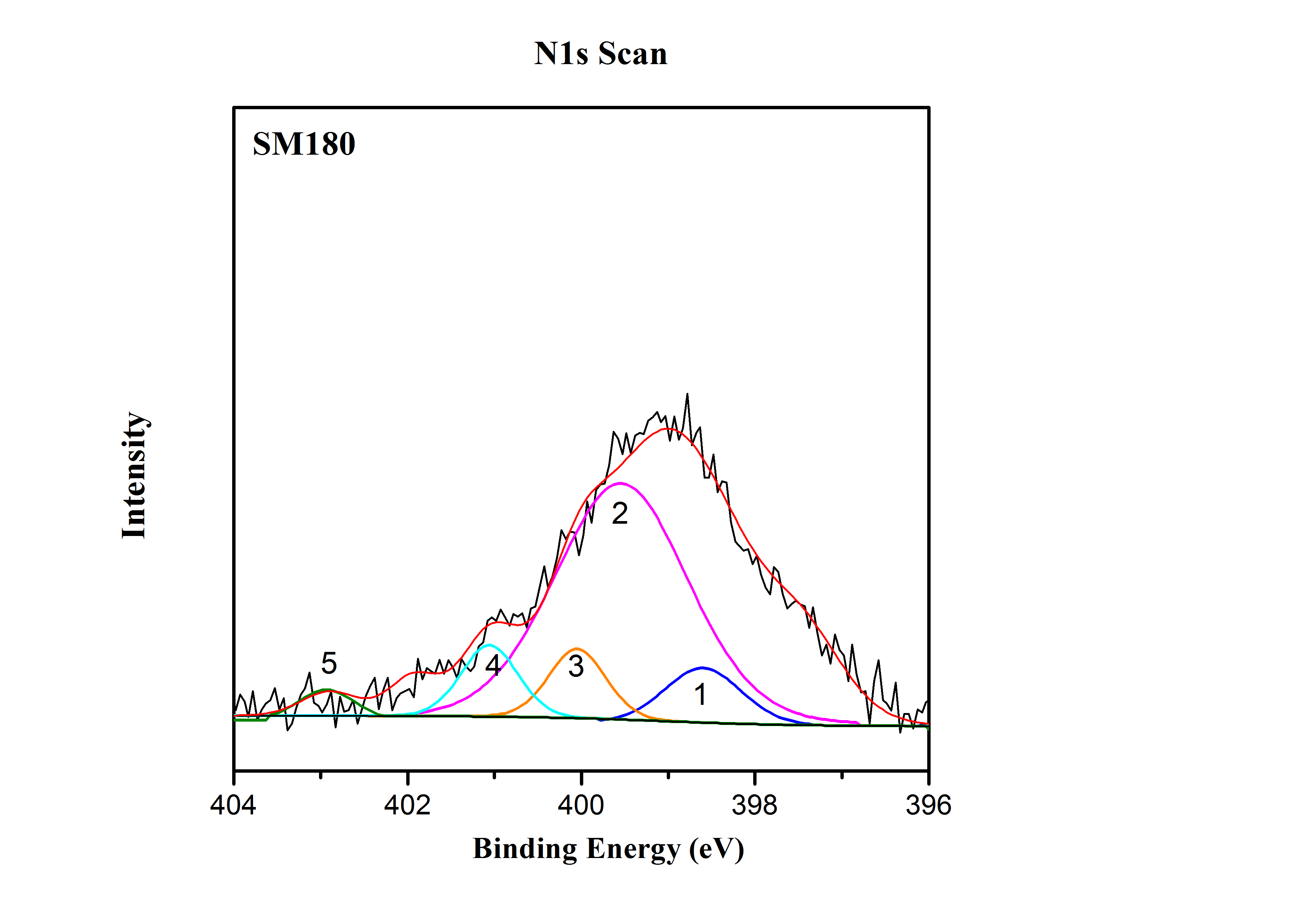

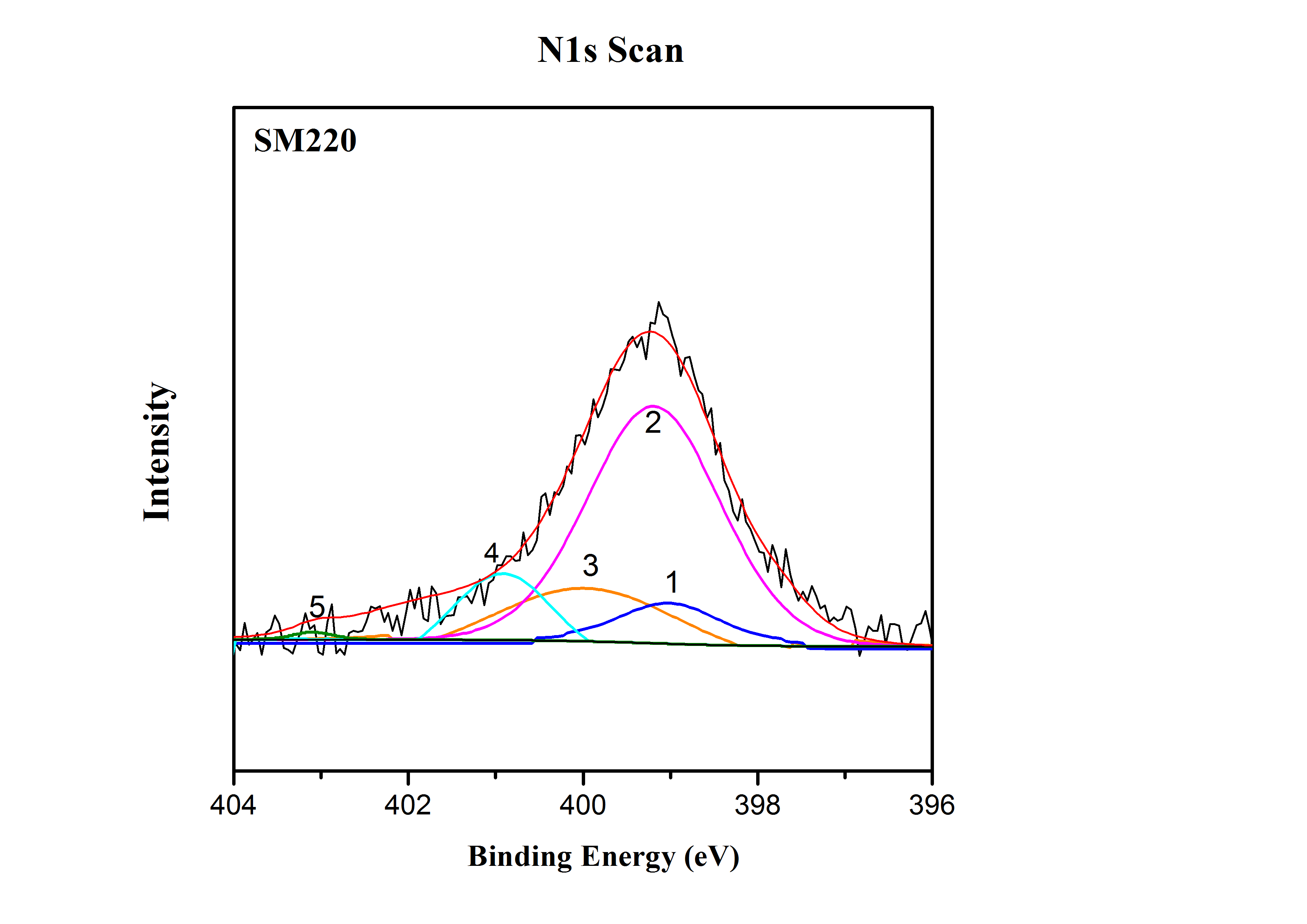
**

(b) (c)

**
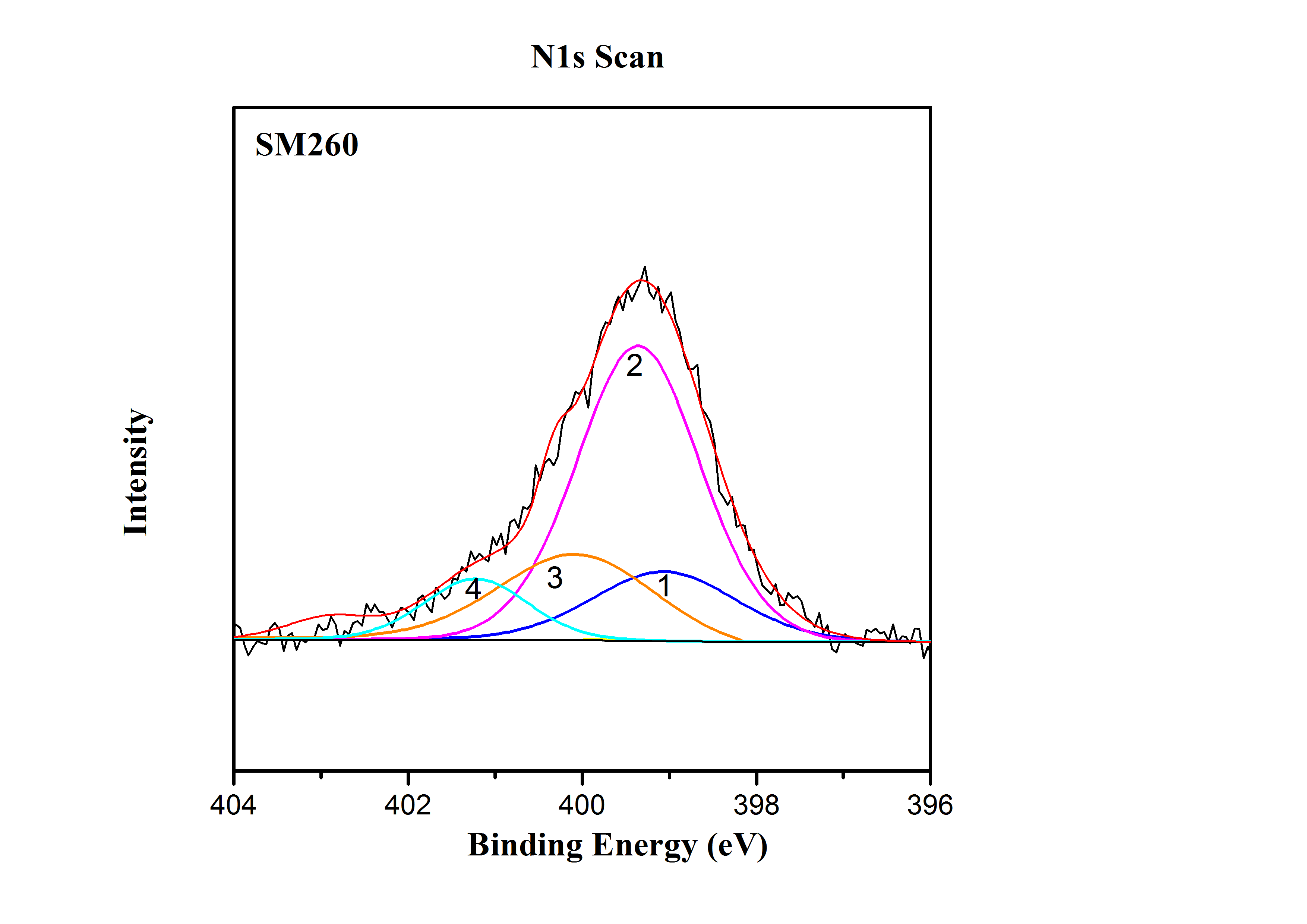

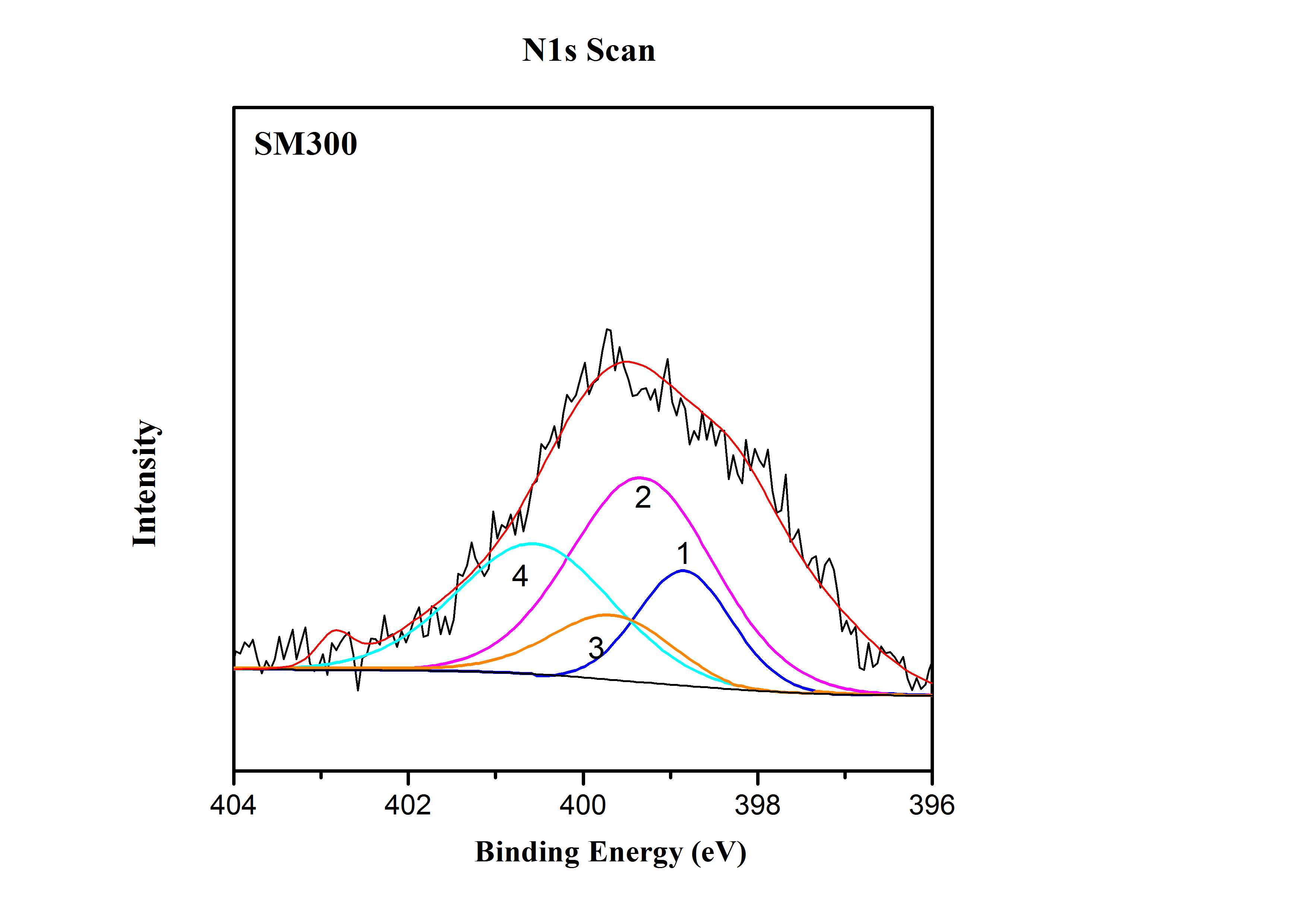
**

(d) (e)

**Fig. A**. N 1S XPS spectra of SM and biochar prepared from thermochemical liquefaction of SM at various temperature


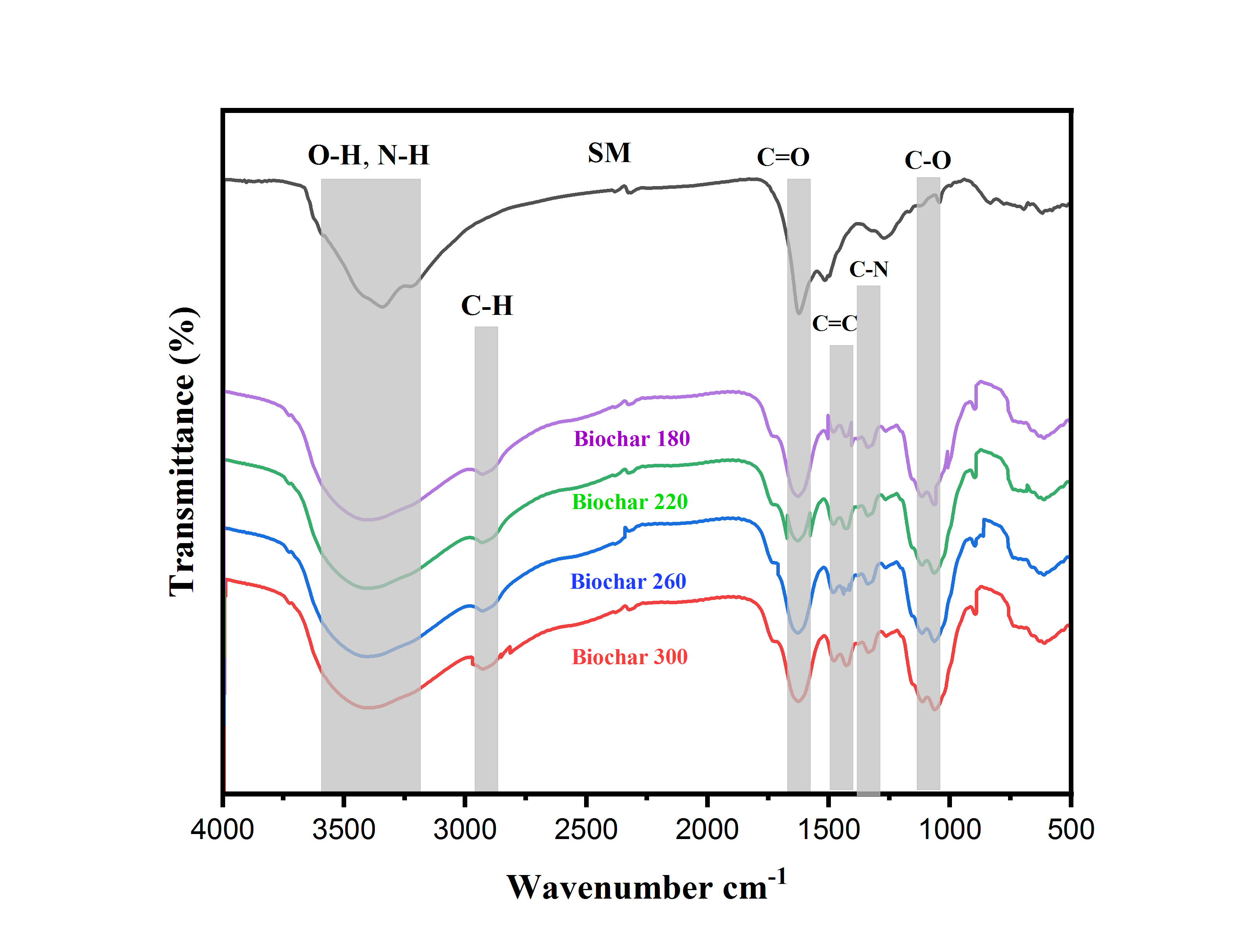


**Fig. B**. FTIR spectrum of SM and biochar.
